# Uses of Bacteriophages as Bacterial Control Tools and Environmental Safety Indicators

**DOI:** 10.3389/fmicb.2021.793135

**Published:** 2021-11-30

**Authors:** Paula Rogovski, Rafael Dorighello Cadamuro, Raphael da Silva, Estêvão Brasiliense de Souza, Charline Bonatto, Aline Viancelli, William Michelon, Elmahdy M. Elmahdy, Helen Treichel, David Rodríguez-Lázaro, Gislaine Fongaro

**Affiliations:** ^1^Laboratory of Applied Virology, Department of Microbiology, Immunology and Parasitology, Federal University of Santa Catarina, Florianópolis, Brazil; ^2^Department of Chemical and Food Engineering, Federal University of Santa Catarina, Florianópolis, Brazil; ^3^Laboratory of Microbiology and Bioprocesses, Federal University of Fronteira Sul (UFFS), Erechim, Brazil; ^4^Contested University (PMPECSA), Concórdia, Brazil; ^5^Laboratory of Environmental Virology, Environmental Research Division, Department of Water Pollution Research, National Research Centre, Giza, Egypt; ^6^Division of Microbiology, Department of Biotechnology and Food Science, Universidad de Burgos, Burgos, Spain; ^7^Centre for Emerging Pathogens and Global Health, Universidad de Burgos, Burgos, Spain

**Keywords:** bacteriophages, food safety, biocontrol, foodborne pathogens, antimicrobial resistance

## Abstract

Bacteriophages are bacterial-specific viruses and the most abundant biological form on Earth. Each bacterial species possesses one or multiple bacteriophages and the specificity of infection makes them a promising alternative for bacterial control and environmental safety, as a biotechnological tool against pathogenic bacteria, including those resistant to antibiotics. This application can be either directly into foods and food-related environments as biocontrol agents of biofilm formation. In addition, bacteriophages are used for microbial source-tracking and as fecal indicators. The present review will focus on the uses of bacteriophages like bacterial control tools, environmental safety indicators as well as on their contribution to bacterial control in human, animal, and environmental health.

## Introduction

Bacteriophages, also known as phages, are prokaryotes viruses, being the most abundant life form, present in all environments and the predominant entities in the sea (Boehme, 1993; Suttle, 2005). Several studies have demonstrated a 1:5 relative abundance between bacteria and bacteriophage (Fuhrman, 1999; Balter, 2000; Rohwer, 2003). They were discovered independently by Twort (1915), who isolated them from *Staphylococcus* spp., and from patients with dysentery. D’Herelle (1926) described bacteriophage as a virus that has the capability to parasitize bacteria (Twort, 1915; Delbruck, 1942). Bacteriophages vary greatly in morphology and replicative characteristics, containing either RNA or DNA, being these parameters currently used by the International Committee on Taxonomy of Viruses (ICTV) for bacteriophage classification (King et al., 2012; Table 1). However, the identification of bacteriophages is difficult since there are no universally conserved markers, unlike e.g., the bacterial 16S rRNA gene (Paul et al., 2002), with only minor parts of bacteriophage genomes being used to determine family specific makers, such as the viral capsid g20 of T4 (Fuller et al., 1998; Marston and Sallee, 2003; Sullivan et al., 2008).

**TABLE 1:** Taxonomy, morphological, and molecular characteristics of bacteriophage groups.

Family	Genus	Nucleic acid	Morphology	Main host
*Leviviridae*	*Levivirus*	ssRNA	Icosahedral	*E. coli*
*Cystoviridae*	*Cystovirus*	dsRNA	Icosahedral	*Pseudomonas* spp.
*Microviridae*	*Phix174microvirus*	ssDNA	Icosahedral	*E. coli*
*Inoviridae*	*Fibrovirus*	ssDNA	Filamentous or rod	*Vibrio* spp.
*Podoviridae*	*P22virus*	dsDNA	Tailed	*S. typhimurium*
*Plasmaviridae*	*Plasmavirus*	dsDNA	Spherical to pleomorphic	*Mycoplasma* spp.

**FIGURE 1:**
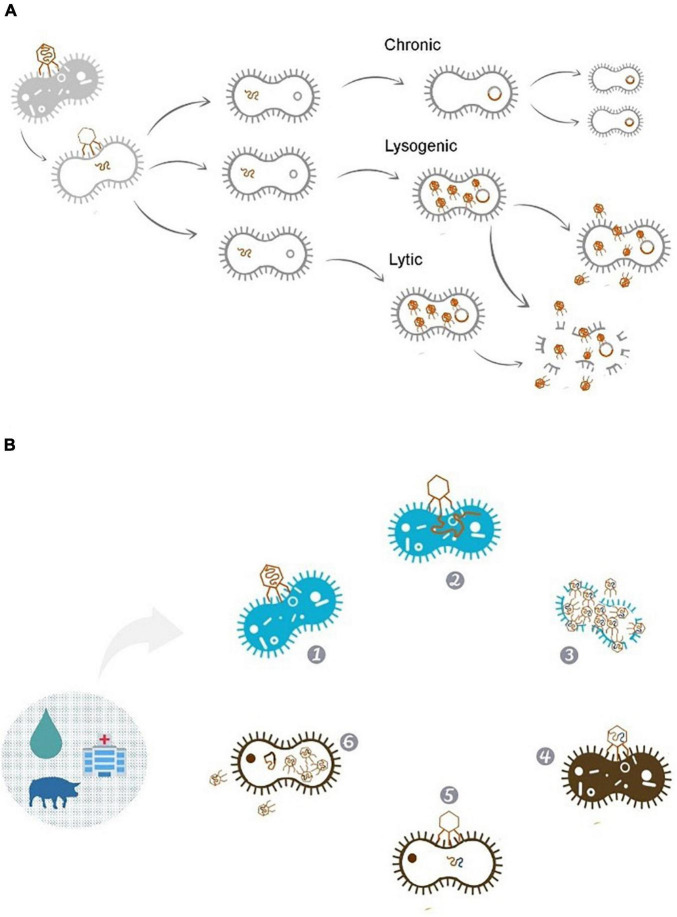
**(A)** Bacteriophage life cycle. Bacteriophages can replicate by lytic, lysogenic or remain in a chronic state. In the lytic cycle of replication the viruses are released from the host after completing their replication. Lysogenic cycle involves the inclusion of genetic material into the genome of host cells, this way phages can contribute to the transferring of tetracycline resistance plasmids to bacterial cells (Pratama and Van Elsas, 2017). This is evidenced considering bacteriophages genomes, which can represent up to 13 and 5% of the *Escherichia coli* O157:H7 and *Salmonella* Newport genomes, respectively (Bobay et al., 2013). **(B)** Phage’s journey through different cell lines in different environments: (1) Bacteriophage adhering to the resistant bacterial host. (2) Insertion of the phage genetic material. (3) New phages carrying resistance genes derived from the infected bacteria, being released into the environment. (4) Adhesion of the new phage on non-resistant bacteria. (5) Passage of resistance genes inserted into the phages genome to the bacteria. (6) After the genes insertion, the bacteria can enjoy the resistance genes acquired while new bacteriophages can be released keeping the journey of resistance genes on the environment.

Bacteriophages can present different life cycles: lytic, lysogenic, and chronic (Figure 1A). Lytic bactériophages, such as T4 and MS2, insert their genetic material inside the bacteria, forcing the cell to produce a large amount of bacteriophage copies. After replication the membrane is then ruptured, releasing the new bacteriophages. Lysogenic bacteriophages (such as T1) possesses an alternative sub-cycle, in which the virus may integrate its DNA in the bacterial genome, becoming non-infectious and replicating together with the bacterial chromosome; the bacteriophage then becomes a prophage, producing new bacteriophage particles under appropriate conditions. Finally, chronic bacteriophages (such as M13) preserve their genome in the bacterial cell, in which the release from the host occurs gradually with less damage to the cell, preserving it longer (Clokie et al., 2011; Cann, 2016; Janczuk et al., 2016). There is an intimate relation between bacteriophages and bacterial cell functions acquisition (Forterre, 1999; Filée et al., 2002, 2003). Bacteriophages can serve as points for genomic rearrangements due to their mosaic nature, with lysogenic bacteriophages even protecting bacteria from lytic infection in certain conditions (Brüssow et al., 2004; Tree et al., 2014; Penadés et al., 2015). While bacterial hosts can benefit from the presence of bacteriophages (as they can express important regulators for adaptation to specific niches by the addition of bacteriophage genes in the cell’s genome) bacteriophages can be involved in the transfer of virulence genes, producing proteins participating in invasion, immune evasion, and toxins related to toxin-mediated diseases (Brüssow et al., 2004; Boyd, 2012; Tree et al., 2014; Penadés et al., 2015).

The specificity of bacteriophage infection allows their application in several areas such as biotechnology, ecology, health and environment (bacterial control), and as environmental monitoring agents (Armon and Kott, 1996; Leclerc et al., 2000; Arredondo-Hernandez et al., 2017; McMinn et al., 2017; Friedman and Lai, 2018; Vandamme and Mortelmans, 2019).

In this review, a vast amount of scientific literature has been reviewed on the application of phage-based products, discussing the benefits and limitations of the use of bacteriophages as bacterial control tools in the health, food, and environmental fields.

## Bacteriophages Application

### Fighting Bacterial Infections

Bacterial infections are a major public health concern worldwide, representing an enormous economical and medical burden with a fatal outcome in a significant proportion of those affected. Dysentery caused by *Shigella* spp., *Salmonella* spp., *Proteus* spp. *Staphylococcus*, *Escherichia coli*, and *Pseudomonas*, usually associated with contamination of food and water, is a serious health problem affecting millions of people annually in the world, with shigellosis, a disease caused by *Shigella* spp., resulting in approximately 600,000 deaths each year (Walker et al., 1990; World Health Organization [WHO], 2017).

Lytic bacteriophages are the main tools for phage therapy, for their capacity to invade the bacterial cell and kill it. Lysogenic bacteriophages could also have an application, the transduction mechanisms could allow the use of bacteriophages as genetic tools to increase bacterial susceptibility to antibiotics; however, this approach has not been widely studied (Lu and Collins, 2009; Edgar et al., 2012). The use of a bacteriophage cocktail for the treatment and prophylaxis of intestinal infections caused by *Shigella* resulted in the patient recovery in 9 days, while antibiotic chemotherapy revealed only a clinical improvement after 29 days (Kutateladze and Adamia, 2008). Similarly, many other bacterial infections can be alternatively treated with bacteriophages, including chronic otitis, chronic infections of wounds, cystic fibrosis-associated pulmonary infections, osteomyelitis, mastitis, chronic infections of the urinary tract, gastrointestinal infections, dental caries, and endodontic infections (Harada et al., 2018; Abedon, 2019).

There is evidence that bacteriophages can be effectively used against bacterial infections, including those that have proved to be resistant to treatments with antibiotics (Abedon, 2019). *Staphylococcus aureus*, for example, is reported to be resistant to methicillin (MRSA), vancomycin (VRSA), and vancomycin-intermediate sensitivity (VISA) (Fadlallah et al., 2015). Some studies have shown that bacteriophage therapy for the treatment of infections caused by such bacteria has been successful. Fadlallah et al. (2015) reported the treatment of corneal abscess caused by VISA using the bacteriophage *S. aureus* SATA-8505 (ATCC PTA-9476).

Although treatment with bacteriophages seems a promising advantage compared to conventional antibiotics and disinfectants, a major drawback of this approach is the need for identification of the specificity range against the pathogenic bacteria prior to starting the bacteriophage treatment and the lack of protocols for testing bacterial susceptibility *in vitro* (Kutateladze and Adamia, 2010). As with antibiotics, if incomplete bacterial elimination by bacteriophages occurs, this could result in the pathogen reemergence (Carlton et al., 2005; Bigwood et al., 2008). A probable explanation could be that bacteria might show a temporal resistance, or that the bacteriophage infection results in high levels of reduction but not a complete elimination of bacteria (Hoskisson and Smith, 2007; Tokman et al., 2016; Moye et al., 2018).

However, contrasting findings of the bacteriophages cocktails effectiveness (compared to “conventional” treatments such as antibodies) were also achieved, with limitations and advantages in the use of cocktails to treat bacterial infections being extensively reviewed (Altamirano and Barr, 2019; Furfaro et al., 2018; Principi et al., 2019). In study conducted by Jault et al. (2019), a cocktail contend 12 bacteriophages was compared to antibody treatment in patients with skin infections, in a randomized control trial. At the end of the study, the conventional treatment with a 1% sulfadiazine silver emulsion cream was still more effective than the cocktail. However, more randomized, placebo-controlled trials must be done in order to have some consensus in dealing with the use of bacteriophages to treat infections.

There are some limitations in the use of bacteriophages for treating human infections. Due to the ability of certain bacteriophages to integrate their genome into the host’s genome, care must be taken when selecting isolated bacteriophages. Some bacteriophages have potential for gene transfer; for instance, the bacterial acquisition of antibiotic resistance genes (ARGs) occurs by transduction, with bacteriophages acting as mobile genetic elements (MGE). Consequently, bacteriophages have been studied as possible vehicles of ARGs, not only as a source, but also as propagators in the environment (Gunathilaka et al., 2017). Bacteriophages containing ARGs are present in a wide range of environments; however, some environmental niches have a greater abundance, such as freshwater or marine environments (Lekunberri et al., 2017a,b; Calero-Cáceres and Luis, 2019). Bacteriophages can be also found in hospital wastewater, yet human-associated viromes rarely charge ARGs (Figure 1B; Enault et al., 2017; Lekunberri et al., 2017a,b).

### Control Tools for Food and Environmental Bacterial Contamination

Foodborne pathogens are a major food safety threat, in 2010 an estimated 2 billion individuals contracted foodborne illnesses, resulting in 1 million deaths around the world (Kirk et al., 2015). Food safety is regarded by the World Health Organization [WHO] (2015) as a major obstacle in human development, especially in developing countries that lack infrastructure and proper environmental health practices to counter the issue. The application of bacteriophages has been proposed as an alternative tool to disinfect food and food-related environments (Pang et al., 2017). The advantage of this method is that bacteriophages kill their bacterial hosts without changing food organoleptic properties (Loc-Carrillo and Abedon, 2011; Perera et al., 2015). Also, bacteriophage low-cost large scale production, self-replicating nature, and low toxicity provide a cheap and safe disinfecting agent for low-income communities, being employed in the former Soviet Union for over 100 years (Skurnik et al., 2007; Abedon et al., 2011; Wójcik et al., 2020).

The United States Department of Agriculture (USDA) approved some products based on bacteriophages as food sanitizers, such as ListShield™, Listex P-100™, SalmoFresh™, and Salmonelex™ (Hagens and Loessner, 2010). The use of a bacteriophage cocktail to inactivate foodborne bacteria like *S. enteritidis* and *S. typhimurium* on the chicken breast has also been proposed (Duc et al., 2018). These bacteriophages have been isolated from environmental sources such as wastewater, sewage, water or food (Pereira et al., 2016). Bacteriophages may also be applied for biofilm control on the food industry, such structures form on surfaces that have been improperly sanitized (Jessen and Lammert, 2003). Outbreaks of bacterial pathogens associated to biofilms in food chain have been related to the presence of *Listeria monocytogenes*, *Yersinia enterocolitica*, *Campylobacter jejuni*, *Salmonella* spp., *Staphylococcus* spp. and *E. coli* O157:H7 (Aarnisalo et al., 2007). In this context, bacteriophages have been suggested as a green strategy for biofilm control, as they may provide a natural, highly specific, non-toxic, feasible approach for biofilm formation control (Grant et al., 2017). Biofilm control using bacteriophages has also been used to prevent dental caries, where the bacteriophages were first isolated from saliva samples and also in biofilm-mediated illness like endodontic disease, which is caused by dental pulp infection (Stevens et al., 2009; Dalmasso et al., 2015). However, it is important to highlight that each bacterial serovar could show different degrees of susceptibility to different bacteriophages (Grant et al., 2017). In addition, it is important to highlight that biofilm control by bacteriophages is mediated by the chemical composition, environmental factors, growth stage and bacteriophage concentration. Additionally, bacteriophage-biofilm interactions depend on the susceptibility of the biofilm cells and availability of receptor sites, where bacteriophage production of polysaccharide-degrading enzymes combined with effective cell lysis may rapidly destroy the biofilm (Simões et al., 2010).

Bacteriophages also show significant potential in the animal production chain such as fish, poultry, shrimps, oysters, sheep, pork and also as additives in food products such as poultry meat and eggs (Moye et al., 2018). They can prevent foodborne pathogens such as *Campylobacter* spp., *E. coli*, *L. monocytogenes*, *Salmonella enterica*, and *Shigella* spp., that are the top five foodborne public health threatening pathogens (Figueiredo and Almeida, 2017; Harada et al., 2018; Kim et al., 2020).

Bacteriophages have shown very effective to control *L. monocytogenes* by the commercial product based on bacteriophages LISTEXP™100 reported a better efficacy against *L. monocytogenes* than nisin and sodium lactate in ready-to-eat (RTE) sliced pork ham (Figueiredo and Almeida, 2017). Chibeu et al. (2013) used a Listeria-specific bacteriophage on the surface of deli meats; a single bacteriophage strain was effective in reducing the numbers of *Listeria* cells (ATCC 19115). The evaluation of LISTEX™P100 as a bacteria controller measured the bacteriophage inactivation using black tea extract and ferrous sulfate and isolation of regrowth bacteria and their control. The result was the reduction of 1.5–2.1 log_10_ CFU/cm^2^ on RTE meat samples by application of 100 μl LISTEXP™100 covering 10 cm^2^ area during 28 days, resulting on 10^7^ PFU/cm^2^ final concentration. A cocktail of bacteriophages can be a more effective approach against a unique species of bacteria, ensuring that resistant bacteria are not selected. The application of the cocktail ListShield™ including six *L. monocytogenes* specific bacteriophages efficiently reduces this pathogen in cheese, smoked salmon, apple slices, and frozen entrees (reduction of 91, 82, 90, and 99%, respectively), without changing the food organoleptic properties (Perera et al., 2015). Similarly, reductions of up to 5 logs of *L. monocytogenes* were observed in various solid foods, such as smoked salmon, iceberg lettuce leaves, sliced cabbage, hot dogs, mixed seafood, turkey meat, and mozzarella cheese brine (Guenther et al., 2009). In fact, the use of a lytic bacteriophage on soft cheese was able to reduce 2 logs of the *Listeria* contamination while maintaining the natural microbial community of the cheese, reinforcing the host specificity of bacteriophages, and in this case the bacteriophage A511 (Guenther and Loessner, 2011).

The number of commercial solutions containing bacteriophages is increasing worldwide, being an emerging industry and field of research (Sulakvelidze, 2013; Vikram et al., 2020). Different examples of bacteriophage applications on food industry are already available: a three-bacteriophage cocktail (containing EC6, EC9, and EC11) was able to reduce *E. coli* contamination; *E. coli* ATCC 25922 and *E. coli* O127:H6 in Ultra High Temperature (UHT) milk at 25°C and under refrigeration temperatures (5–9°C) (McLean et al., 2013). The cocktail EcoShield™ was able to reduce 2 logs of *E. coli* O157:H7, 30 min after administration on leafy greens under packaging storage (Boyacioglu et al., 2013). Magnone et al. (2013) verified the disinfection of *E. coli*, *Salmonella* and *Shigella* from broccoli, cantaloupe and strawberries, with the use of commercial bacteriophage cocktails (EcoShield™, SalmoFresh™, and ShigActive™) being as effective or even more than chlorine wash. *Salmonella* is a major threat for the food industry and the most common zoonotic foodborne pathogen isolated from livestock (Jajere, 2019). The bacteriophage FO1-E2 was able to reduce the levels of *Salmonella* contamination on milk and mixed seafood for 24 h, remaining undetectable at 8 and 15°C (Guenther et al., 2012). Similarly, bacteriophage wksl3 was also able to decrease by 3 logs *Salmonella* contaminations on chicken skin (Kang et al., 2013). Likewise, some bacteriophage cocktails for *Salmonella* control are also available. The commercial formulation SalmoFresh™ was able to reduce 2–3 logs of *Salmonella* on lettuce and sprouts, showing greater reduction (2.7–3.8 logs) when associated with chlorinated water (Zhang et al., 2013). An outstanding biocontrol activity was seen with the use of a bacteriophage cocktail composed by LPSTLL, LPST94, and LPST153, being able to reduce >5.23 log viable *Salmonella* counts on biofilm grow in microtiter plates and steel chips, for 72 h. The same bacteriophages combination was also able to completely reduce the *Salmonella* inoculum on chicken breast and milk (Islam et al., 2019). Table 2 summarizes the bacteriophages commercially available and the conditions for its applicability on treatment against different bacterial agents.

**TABLE 2:** Current commercial products containing bacteriophages and conditions for use.

Host	Bacteriophage	Dose	Treatment time	Matrix	Reduction log	References
*Listeria monocytogenes*	LISTEX™ P100	10^7^ PFU/cm^2^	30 min, 1, 2, 3, 7, 10, 14, 20, and 28 days	Roast beef and cooked turkey	2 log_10_ CFU/cm^2^	Chibeu et al. (2013)
	FWLLm1	2.5 × 10^7^ PFU/cm^2^	24 h	Ready-to-eat chicken breast roll	2.5 log_10_ CFU/cm^2^	Bigot et al. (2011)
	P100/A511	3 × 10^8^ PFU/g	6 days	Hot dogs (sausages), cooked and sliced turkey breast meat (cold cuts), smoked salmon, mixed seafood (cooked and chilled cocktail of shrimp, mussels, and calamari), chocolate milk (pasteurized, 3.5% fat), mozzarella cheese brine (unsalted pasteurized whey from plastic bag containers containing fresh mozzarella cheese), iceberg lettuce (leaves), and cabbage (sliced fresh leaves)	1–3 log_10_ CFU/cm^2^	Guenther et al. (2009)
	ListShield™	10^9^ PFU/mL	0, 2, 5, and 7 days	Fresh-cut melons and apples	3.5 log_10_ CFU/cm^2^	Leverentz et al. (2001)
*Salmonella nteritidis*	SJ2	10^8^ PFU/mL	24 h	Raw and pasteurized milk cheeses	1–2 log_10_ CFU/cm^2^	Modi et al. (2001)
	PHL 4	10^10^ PFU/mL	24 h	Poultry carcass	3 log_10_ CFU/mL	Higgins et al. (2005)
*Salmonella typhimurium*	Felix-O1	5.25 × 10^6^ PFU	24 h	Chicken frankfurters	2 log_10_ CFU/g	Whichard et al. (2003)
	SalmoFresh	10^8^ PFU/mL	5 h	Ready-to-eat chicken products	2 log_10_ CFU/mL	
*S. enteritidis* and *S. typhimurium*	wksl3	2.2 × 10^8^ PFU/mL	1, 2, 3, 5, and 7 days	Chicken skin	3 log_10_ CFU/mL	Kang et al. (2013)
	SalmoFREE	10^8^ PFU/mL	36 days	*In vivo*–chicken production	3 log_10_ CFU	Clavijo et al. (2019)
	Salmonelex™	10^9^ PFU/mL	24 h	Ground beef and ground pork	1.1 and 0.9 log_10_ CFU/g	Yeh et al. (2017)

### Indicators of the Presence of Foodborne Pathogens

Bacteriophages have been suggested as an alarm system in food and environmental microbiology and epidemiology since they generally fit the indicator criteria of pollution. Bacteriophages can be used as fecal indicators or microbial water quality bioindicators as an early warning of contamination by sewage, and as an efficiency marker of water or wastewater treatment (Yahya et al., 2015). This can be attributed to the bacteriophage response to the presence of pollutants, they are characteristic to adsorb to solid particles in the environment, and also due to some limitations of traditional indicators for public health such as fecal coliforms, *E. coli* and enterococci (Armon and Kott, 1993; Ashbolt et al., 2001; Jofre et al., 2016; McMinn et al., 2017).

Somatic coliphages are more persistent than traditional indicators, being also more resistant to sludge treatments, particularly when adsorbed to surfaces (Martín-Díaz et al., 2020). Many authors highlight the use of bacteriophages as indicators not only for enteric pathogenic bacteria, but also for enteric viruses such as human noroviruses, adenoviruses, and rotaviruses (Guelin, 1948; Dutka, 1990; Cornax et al., 1991; Kott, 1992; Armon and Kott, 1996; Leclerc et al., 2000; Arredondo-Hernandez et al., 2017; McMinn et al., 2017). This characteristic is due to the wide stability of phages in waste, water, soils and residues, with F-specific phages and somatic coliphages being the most used for monitoring water quality (Grabow, 2001; Sinton et al., 2002).

One of the challenges with bacteriophage application is related to bacteriophage-host interaction, which could vary depending on exposition temperature, where greater bacterial reductions are associated with higher temperatures (Tomat et al., 2013). The use of bacteriophages on wastewater treatment systems is based on their lytic capacity, which is a useful tool for the removal of human and animal pathogenic bacteria from wastewater or applied as an indicator for the presence of bacteria in wastewater treatment systems (Stefanakis et al., 2019). MS2 bacteriophages have been proposed to be suitable as operational monitoring indicators as established by guidelines of Australia, due to resistance to variation of pH and temperature (Amarasiri et al., 2017). Other applications of bacteriophages in the improvement of environmental quality are based on their survival in the environment, and soil percolation to control pathogenic bacteria in underground water (Ye et al., 2019). However, there are still some challenges for the use of bacteriophages in wastewater treatment: a high bacteriophage dosing must be used for a successful application and the use of polyvalent bacteriophages with a wider host variety could result in the reduction of beneficial bacteria. The bacterial analysis of the system is a basic requirement for bacteriophage application, as the bacterial population can change in the wastewater treatment plant (Jassim et al., 2016).

## Challenges, Concerns and Trends in the Use of Bacteriophages for Environmental Health Purposes

Although a worldwide acceptance of bacteriophages as environmental agents is not yet achieved, bacteriophage-based technologies in the environmental field are still being developed. Besides being employed as monitoring agents, or by directly controlling pathogens, bacteriophages have demonstrated promising results in agricultural microbiome modulation, increasing crop production by infecting crop detrimental bacteria in leaves and soil (Jones et al., 2012; Ye et al., 2019). Plant-soil microbiome modulation by bacteriophages was even related to an increase in ammonium concentration, likely through lysis of certain bacteria and overall community shifting (Braga et al., 2020). The use of bacteriophages on plant soil was referred to as a safer and more reliable antibacterial agent than antibiotics, in which the exaggerated use of these chemicals was related to the development of ARGs and inhibition of soil phosphatase activity (Liu et al., 2009; Zhang et al., 2017; Sun et al., 2019).

Similar to soil applications, bacteriophages appears to have a low environmental impact in fish farming plants compared to “traditional” methods such as antibodies, as it is necessary a continuous application since seawater is considered a reservoir of antibiotic resistance bacteria (Almeida et al., 2009; Alves et al., 2014; Hatosy and Martyiny, 2015). Even though bacteriophages can be considered as highly flexible and cheap tools, some drawbacks concerning the safety and overall effectiveness of the phage product may hinder their implementation as a widely accepted technology (Payne and Jansen, 2003). Bacteriophages can increase bacteria pathogenicity and fitness by transferring toxin and environmental resistance encoding genes to nearby bacteria, essentially creating genetic hazards in the area of application (Colomer-Lluch et al., 2011; Feiner et al., 2015). Besides bacteriophage-induced resistance, the bacteria may also become resistant to the virus activity through spontaneous mutations or through adaptive immunity *via* the CRISPR system (Labrie et al., 2010). Another possible major drawback in bacteriophage application is the potential disruption of the local microbiome, consequently favoring the development of harmful bacteria or health problems associated to a microbiome disbalance. Bacteriophage application has been tied to microbiome dysbiosis in humans, and can be related to the subsequent development of intestinal and mental diseases (Norman et al., 2015; Tetz et al., 2018). Microbiome disruption was also related to the development of diseases in both livestock and plants, therefore an improper bacteriophage-based product (i.e., bacteriophages that may infect healthy microbiome) may also potentially harm animal and plant farming production (Meaden and Koskella, 2013; Zeineldin et al., 2018; Lei, 2020).

In sight that bacteriophages may persist in food production plants due the virus high stability, potentially creating a genetic hazard in such facilities, the adoption of strategies for the use and manipulation of bacteriophages are required to counter bacteria resistance and achieve successful pathogen control (Hungaro et al., 2013; Chaturongakul and Ounjai, 2014; Fister et al., 2016, 2019). In this regard, practices that reduce the probability of bacteriophage resistance occurrence must be preferred, such as a two-stage self-cycling or a cellstat process (García et al., 2019). Bacteriophage cocktails have been also been employed as a way to counter bacterial bacteriophage resistance, in this strategy the bacteria would be unable to adapt (or have their viability greatly reduced) to the different infective dynamics of each virus, however, knowledge about the cocktail pharmacodynamic is required to achieve a multi-targeting system against the same bacterial strain (Abedon et al., 2021).

Aside from ARGs screening and mapped host targeting, the phage product must be suitable to the external factors present in the area of application, being resistant to the pH, temperature, UV radiation, salinity and ionic profile of the environment (Jończyk et al., 2011; Zaczek-Moczydłowska et al., 2020). In addition, the criteria for bacteriophage use in food and the environment, such as minimum exposure time, minimum effective dosage and characterization of animal local application must be established to achieve the expected therapy result while avoiding potentials drawbacks such as the presence of inhibitory compounds like antibodies, whey proteins or bacteriocins (Abedon, 2012; Vongkamjan et al., 2013; Ly-Chatain, 2014).

Special regards covering the bacteriophage properties are also advisable for an optimal and highly scalable confection of the final viral product, being of special relevance in extensive environmental applications. Bacteriophage production is directly related to the characteristics of the bacterial host (e.g., metabolic activity, growth rate, stage in cell life cycle, and abundance of bacteriophage receptors on cell surface), and the bacteriophage attributes (e.g., lysis time, burst size, and adsorption rate) (Agboluaje and Sauvageau, 2018). In addition, the initial multiplicity of infection (MOI), pH, aeration rate, presence of ions or cofactors, agitation and medium composition may also influence the outcome of infections, thus affecting bacteriophage production (Agboluaje and Sauvageau, 2018). Therefore, a full characterization of the virus and host synergy is highly advisable for easy escalation of the phage product (García et al., 2019).

With advances in molecular biology the engineering of bacteriophage particles allows a selected virus (favorited due desirable characteristics to the target therapy, such as host range and replicative potential) to be further enhanced through genetic modifications, removing undesirable viral properties that could hinder the application of the bacteriophage product as a safe and reliable object (Górski et al., 2018). Genetic engineering of phage products was able to remove toxin encoding genes and increment the virus stability in low pH environments, enhancing the functionality and removing safety hazards of the final viral product without requiring the selection of new bacteriophage strains (Nobrega et al., 2016; Park et al., 2017).

Although bacteriophages present certain safety drawbacks, largely due to negligence of mapping the product properties, bacteriophages are still considered safer than chemical treatments in environmental and food processing plants treatments applications (Meaden and Koskella, 2013; Zaczek et al., 2014). Bacteriophages stand as cheap and highly flexible structures, being able to be selected and edited for different approaches (Farr et al., 2014; Sunderland et al., 2017). Most of the research on bacteriophages has highlighted the potential for *in vitro* applications, and the number of scientific publications has increased in the last decades due to the potential use of bacteriophages in a broad spectrum of applications. In health sciences, bacteriophages are a promising approach in the fight against antibiotic-resistant bacteria, and, in the food chain, they could be a safe alternative for the control of foodborne pathogens. However, to guarantee effectiveness, a detailed understanding of the interaction between bacteriophages and the hosts is needed, considering restrictive criteria for their use to minimize their negative impact on food and food-related environments.

## Author Contributions

PR directs the first version of the manuscript. GF and DR-L revised the first version of the manuscript and wrote the final version of the manuscript. The rest of the authors gave fundamental contributions to the first version of the manuscript. All authors contributed to the article and approved the submitted version.

## Conflict of Interest

The authors declare that the research was conducted in the absence of any commercial or financial relationships that could be construed as a potential conflict of interest.

## Publisher’s Note

All claims expressed in this article are solely those of the authors and do not necessarily represent those of their affiliated organizations, or those of the publisher, the editors and the reviewers. Any product that may be evaluated in this article, or claim that may be made by its manufacturer, is not guaranteed or endorsed by the publisher.
